# Quantitative Evaluation of the Movement Distance of Deep Fascia and Change of Muscle Shape Related to Chain Response in Fascia Tissue of Lower Limb

**DOI:** 10.3390/life11070688

**Published:** 2021-07-14

**Authors:** Kazuyuki Sugawara, Mitsuhiro Aoki, Masahiro Yamane

**Affiliations:** 1 EzoReha Co., Ltd., Nishi-ku, Sapporo 01107, Japan; 2 Department of Physical Therapy, Graduate School of Rehabilitation Science, Health Sciences University of Hokkaido, Tobetsu-cho, Ishikari-gun 01303, Japan; 3 Department of Physical Therapy, Health Science University Hospital, Kita-ku, Sapporo 01102, Japan; m-yamane@hoku-iryo-u.ac.jp

**Keywords:** tension response of deep fascia, ultrasound imaging, passive movement of toe and ankle joint, TFL, G-med

## Abstract

By using ultrasonography, we measured the longitudinal movement distance of the deep fascia (LMDDF), change of the pennation angle (PA) and muscle thickness (MT) in both the tensor fasciae latae muscle (TFL) and the gluteus medius muscle (G-Med) during passive movement of the toes/ankle joints. 21 right lower limbs of 21 healthy males were evaluated in this study. We measured the LMDDF of the TFL and G-Med by measuring distance between the designated landmark on skin and the intersection of the major deep-fascia (D-fascia) and the fascial bundle. We also measured change of the PA and MT of both muscles. Additionally, we also measured the reliability of the measurement and the measurement error. The measurement was performed during three manual positions on the toes/ankle; manual holding of the toes and ankle joint in neutral, toes flexion and ankle plantar flexion/inversion position, toes extension and ankle extension/valgus position. The existence of muscle contraction of both the muscles during passive motion was monitored by active surface electrodes. This study confirmed mobility of the D-fascia in which the TFL’s D-fascia moves and change of muscle shape in the distal direction during no muscle contraction due to passive movement. This fact suggests the possibility that passive tension on fascia tissue of the ankle extends to the proximal part of the limb, i.e., to the D-fascia of the TFL.

## 1. Introduction

Since the year 2000, several articles and books have appeared in the medical and physical therapy fields that target membranes and fascia tissues (fascia) as a treatment [1,2]. The deep fascia (D-fascia) is capable of distinguishing two major types of fascia, aponeurotic fascia and epimysial fascia, by their thickness and relationship to the underlying muscles [3]. Aponeurotic fascia involves collagen fiber bundles aligned along the long axis of the limb. As a result, both longitudinally and obliquely, the D-fascia functions like a tendon to transmit force along the limb [4].

According to anatomical findings, Stecco A et al. [5] confirmed that the gluteus maximus muscle (G-max) in all six cadavers is connected to the iliotibial ligament, the femoral fascia, and the tensor fasciae latae (TFL), proving that the femoral fascia provides a small origin from multiple muscles of the thigh, explaining the transmission of forces from the G-max to the peroneal knee. Wilke J et al. [6] reported on the connection between the iliotibial ligament and the fascia of fibularis longus and inferred a mechanical connection between the iliotibial ligament and the lower leg fascia. These studies have demonstrated the “anatomical continuity” of the long axis of the limb by the fascia and the development of the fascia from superficial to deep by anatomical studies, and their importance has been proposed. Ultrasound imaging can provide real-time, non-invasive images of skeletal muscle shape and myofascial tissue during muscle contraction and joint movement [7,8]. However, the D-fascia and perimysium are dense connective tissues, and because of the dense interfibrillar spaces between the fibers, they are linearly depicted as high echoes, and the D-fascia layer can be easily distinguished [9].

Ultrasound makes in vivo morphological evaluation feasible both in real-time and non-invasively. B-mode ultrasound has been used to evaluate the pennation angle (PA) in muscle thickness (MT) measurements during isometric contraction [10,11]. Ichikawa et al. [12] measured the distance moved by the D-fascia by identifying the contact points between the D-fascia in the superficial layer and the D-fascia in the deep layer and the PA using ultrasound imaging, and clarified that the measurement of the distance moved by the D-fascia is clinically applicable. In addition, Hodges P W et al. [13] investigated the relationship between measurements of PA and MT and muscle contraction values using an ultrasound imaging. However, in vivo measurements using ultrasound systems are prone to errors, and it is essential to confirm the reliability of the evaluation method when considering the evaluation of muscle tissue and fascia using ultrasound systems.

Absolute reliability is a method to determine which type of error is contained in the measured value and to what extent. It is a method to clarify the “true change” by indicating the “range” of error contained in the measured value in the same unit (mm, °) as the measurement method, rather than the “coefficient” of relative reliability [14]. Bland–Altman analysis and minimal detectable change (MDC) are two methods to clarify these issues. The MDC95 indicates the limit at which two measurements are due to measurement error, and if the difference between one measurement and the other is greater than the MDC95, a true change can be judged to have occurred [15].

In order to examine the transmission of mechanical tension by the D-fascia, the effects of biological responses and mechanical stimuli caused by mono- and biarticular muscles must be considered. The characteristics affected by these monoarticular and biarticular muscles and the effect of sliding of the D-fascia tissue due to the contraction of these muscles must be eliminated. Therefore, there has been no report on the mechanical tension transfer associated with the anatomical continuity through the fascia caused by the movement of distant joints and muscles in the limb.

The purpose of our research is to understand the transmission of force across multiple joints beyond the influence of biarticular muscles {“distal to proximal force transmission” or “proximal to distal force transmission”} as a “response caused by the anatomical continuity of the deep fascia”, and agreeing with the results of fascia’s connection model [16,17,18,19] and cadaveric anatomical studies [5,6], we define the above transmission of tension as a “tension response of the deep fascia” and examine its response.

The hypothesis of this study was that during passive movement of the ankle joint, changes would occur in the movement of the deep fascia and muscle shape of the G-med and TFL.

## 2. Materials and Methods

### 2.1. Participant

#### 2.1.1. Reliability Considerations

Before conducting this experiment, the reliability of the ultrasonic measurement method of this study was examined and measurement errors were measured on 11 subjects with 11 limbs. The mean age (range) was 26 years (21–31 years), and the mean height and weight (standard deviation: SD) were 171.6 (3.60) cm and 66 (4.52) kg, respectively.

#### 2.1.2. Verification Experiment of D-Fascia Response

This study was approved by the Ethical Review Committee of the Department of Rehabilitation Science, Health Science University of Hokkaido on 13 March 2008 (Approval No. 17R069063). Participants were asked to give written informed consent regarding the content of the experiment, protection of personal information, physical and mental burden of the experiment, and medical compensation for adverse events. The subjects were 21 healthy adult male volunteers with no previous orthopedic history and a mean age (range/SD) of 24 years (21–31 years/4.1). The mean (SD) of height and weight were 171.8 (6.43) cm and 66.9 (7.15) kg, respectively (Table 1). Exclusion criteria were those with a history of lower extremity disease, wounds causing restricted sliding of the skin or soft tissues, burns, and neurological findings. AGrubbs–Smirnov rejection test was performed for each measurement of body information, and no outlier was found. 

### 2.2. Measurement Methods

#### 2.2.1. Measurement Position

**Figure 1:**
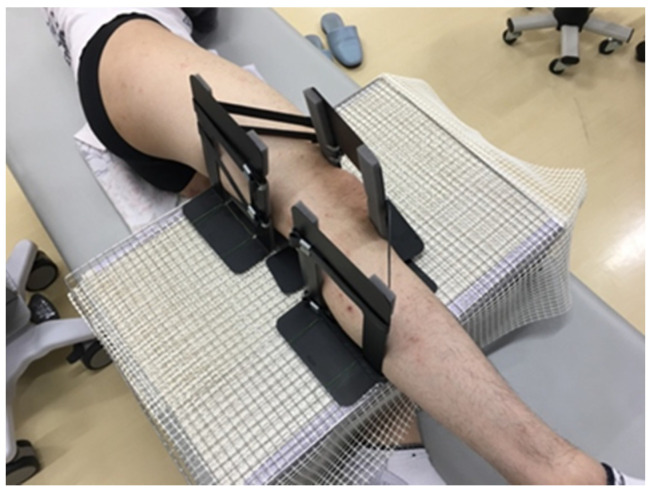
Knee Clamping Device. A knee immobilization device was fabricated to hold the knee joint in the extended position. In order to avoid inhibiting the mobility of the deep fascia due to the compression of the lateral part of the lower limb on the test side, one control plate was placed in front of the knee joint, and two control plates were placed distal to the thigh and proximal to the lower leg to immobilize the lower limb.

A horizontal measuring instrument (Blue Level Digital 450 mm, Shinwa Measurement Co., Ltd., Niigata, Japan) was placed on the outer thigh of the test side (right lower limb) to confirm the horizontal position of the test side lower limb (to define the hip abduction angle.). The knee joint on the measurement side was fixed using a three-point knee joint fixation device (Figure 1), that was created to fix the posterior surface of the thigh proximal to the knee joint, the posterior surface of the lower leg distal to the knee joint, and the anterior surface of the knee joint including the patella, to stabilize and hold the knee joint in the extended position. When fixing the knee joint, care was taken to avoid pressure on the long axis of the soft tissues on the outside of the lower extremity related to the measurement site.

**Figure 2:**
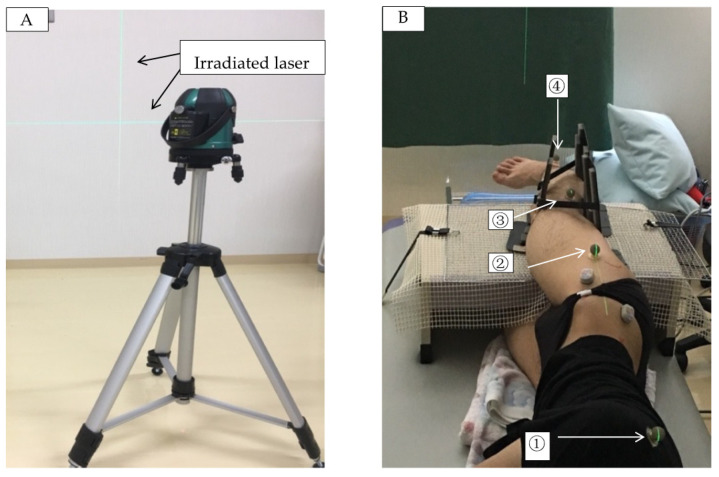
(**A**) Line Laser Marker. Line Laser Marker and irradiated laser beam. (**B**) Line laser beam irradiated on the marker. The line laser beam was irradiated from the head side of the subject to the reflective marker and the posture was adjusted. The markers were placed at (1) the center of the scapular spine, (2) the greater trochanter, (3) the lateral epicondyle of the femur, and (4) the external capsule. The right lower extremity was positioned in a straight line, and the knee and hip joints were monitored to prevent flexion.

For pelvic fixation, the non-measured side (left lower extremity) was fixed at the sacroiliac joint relative to the hip in 90° flexion, and then fixed to the treatment table using a non-stretch belt to inhibit rotation of the trunk and pelvis. In order to eliminate errors in the measurement limb positions in the sagittal plane of the trunk, thighs, and lower legs during the experimental task, a line laser was drawn parallel to the treatment table using a line laser aligner (VLG-2X, VOICE: HEM LLC, Tokyo, Japan). During measurement, reflective markers were attached to the center of the scapular spine, adductor pollicis brevis, lateral femoral epicondyle, and external capsule of the scapula on the test side, and the line laser was superimposed on the reflective markers for alignment in the sagittal plane (Figure 2A,B). The alignment was confirmed for each measurement, and the line laser was constantly irradiated and postured during the entire measurement period so that it remained constant for each condition and measurement.

#### 2.2.2. Measurement by Ultrasonic Equipment

A B-mode probe of an ultrasound system (SONIMAGE HS1, Konica Minolta) was used for ultrasound imaging. To observe the D-fascia on the TFL, the probe was placed along the long axis of the muscle fibers at 2–3 cm ventral and 3–4 cm caudal to the greater trochanter, and the intersection of the D-fascia in the superficial layer and the D-fascia in the deep layer of the TFL was identified. The intersection of the D-fascia in the superficial layer and the D-fascia in the deep layer of the TFL was confirmed.

After confirming the measurement point, an aluminum tape was attached to the distal part of the muscle so that a part of the tape could be visualized on the image. The tape portion is shown as a shadow (black) in the image because of the attenuation of ultrasound transmission. 

**Figure 3:**
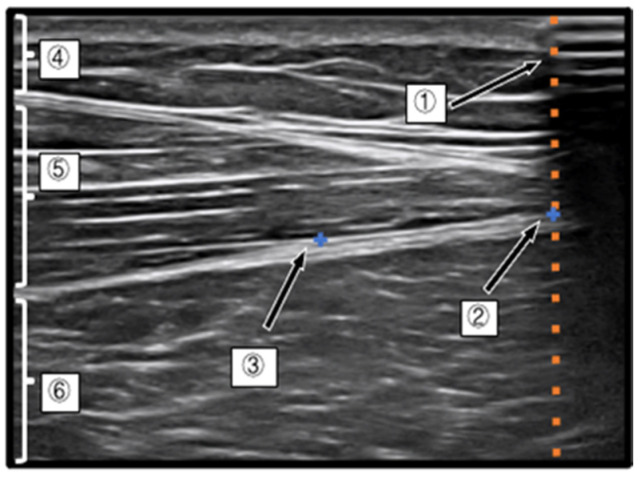
Ultrasound system image of deep fascia of TFL. (1) The area where the ultrasound was shaded by the aluminum tape, (2) the intersection of the shaded area of the aluminum tape and the deep fascia of the TFL, (3) the angle between the deep fascia and a muscle fascicle, (4) the subcutaneous tissue, (5) the TFL, and (6) the vastus lateralis muscle. The left side of the photograph shows the proximal lower extremity, and the right side shows the distal lower extremity.

The landmark (axis of movement) of the D-fascia used to measure the longitudinal movement distance of the D-fascia (LMDDF) of the TFL by ultrasound was identified as the intersection point between the intramuscular muscle fascicle of the TFL and the D-fascia of the TFL. Then, the point at which the examiner could confirm the same landmark six times consecutively was adopted (Figure 3).

**Figure 4:**
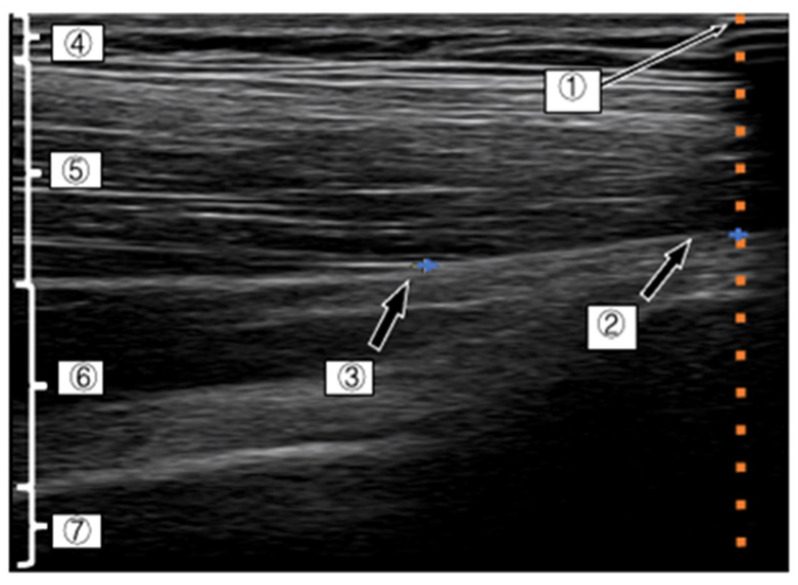
Ultrasound system image of deep fascia of G-med (1) The area where ultrasound was shaded with aluminum tape, (2) the intersection of the shaded area of the aluminum tape and the deep fascia of the G-med, (3) the angle between the deep fascia and a muscle fascicle (4) the subcutaneous tissue, (5) the G-med., (6) Gluteus minimus and (7) the iliac crest. The left side of the photograph shows the proximal leg and the right side shows the distal leg.

For the observation of the D-fascia of the G-med using ultrasound images, we referred to the report by Mitomo et al. [20] and used the myotendinous transition of the middle fibers of the G-med as the measurement site (the probe was placed at the greater trochanter on the line connecting the greater trochanter and the apex of the iliac wing), and the intersection point between the muscle bundle of the G-med and the D-fascia, which was most clearly depicted, was confirmed and used as a landmark. The point at which the examiner could confirm the same landmark after six consecutive readings was then adopted (Figure 4).

After confirming the measurement point of the G-med, aluminum tape was applied to the distal part of the muscle so that a part of the tape could be depicted on the image. The measurements on the ultrasound system in this study were performed by one examiner A (10 years of clinical experience), and several volunteers were trained to perform the measurements in this study in advance. In this study, intra-class correlation coefficient (ICC) and Bland–Altman analysis was used to measure the reliability of ultrasound measurements. 

#### 2.2.3. Electromyography

The electromyogram was measured using a surface electromyograph (cordless active electrode picker, Nihon Kohden) was used. Surface electromyography was performed on the TFL and the G-med as the test muscle. All tested muscles were on the right side. The surface electrodes were placed along the muscle belly 2 cm below the superior anterior iliac spine for the TFL, referring to the method of Shimono et al. [21] In the G-med, the electrodes were placed along the muscle belly in the proximal 1/3 of the line connecting the greater trochanter and the uppermost part of the iliac crest. In order to reduce the noise of the surface EMG, the surrounding AC current was blocked, the skin was disinfected and cleaned to reduce the impedance of the skin, and the electrode surface was cleaned to confirm the EMG of the muscle in a static state. Electromyography is used to demonstrate the inactivity of the TFL and G-med muscles during passive ankle motion.

#### 2.2.4. Measurement Task

The measurement three tasks were as follows. In the passive motion: 1. Ankle joint plantar flexion and dorsiflexion 0°, toe flexion and extension 0° (Resting position). 2. Flexion of the toes to flexion and inversion of the ankle joint (Flexion position). 3. extension of the toes, extension of the ankle joint, and valgus position (Extension position). The angles of passive toe flexion, ankle plantar flexion, and inversion were defined as the maximum range of motion of the subject. The passive toe extension, dorsiflexion and external rotation of the ankle joint were also defined as the maximum range of motion of the subject.

After confirming the measurement points by their resting position, the order of flexion position and extension position and the order of the measured muscles were randomized beforehand and the measurements were performed. 

The external force applied to the foot by the examiner is controlled by a hand-held dynamometer (Sakai Medical, Tokyo, Japan) at a pressure of 5 kgf. In order to define the position for applying pressure, the position for applying external force is defined as the top of the second to fourth metatarsal bones (plantar side) 10 cm (0.1 m) distal to the sole on the extension of the lower end of the endocarp after maximum extension of the toes in case of the extension position {external force (0.5 kgfm) = 5 kgf × 0.1 m}. In order to specify the external force applied during the flexion position, the position where the external force was applied was specified as 10 cm (0.1 m) distal to the sole of the foot on the extension of the lower end of the endocarp, over the second to fourth metatarsals (dorsal part). {external force (0.5 kgfm) = 5 kgf × 0.1 m} 

The examiner fixed the limb position for 5 s with the target external force. Electromyographic waveforms were measured during the 5 s of holding and still images of ultrasound images were taken at 3 s during the 5 s task. Each muscle was measured three times for each task, for a total of nine measurements per muscle.

#### 2.2.5. Data Analysis

The ultrasound image data taken during the resting position, flexion position, and Extension position joint movements were imported into a digital measurement software (Image J, NH, Wisconsin) to measure the length between the following index points, each PA, and MT necessary to measure the LMDDF.

##### Measurement of the Index Point of the D-Fascia and Calculation of the Movement Distance

**Figure 5:**
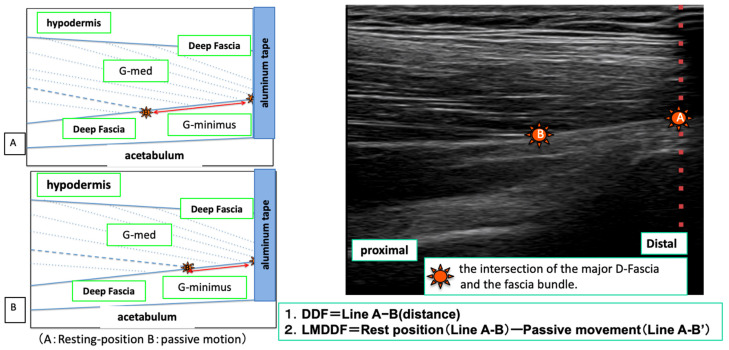
Measurement of the distance of deep fascia (DDF) movement of G-med. (**A**): resting position, (**B**): during passive movements).

For the measurement of the index point of G-med using the ultrasound system, the subject was asked to set the index point (point A) of the shaded image of the aluminum tape depicted in the resting position image and the index point (point B) of the intersection of the D-fascia and the intramuscular muscle fascicle as landmarks, and still images were taken three times. We measured the distance of “points A-B” from the obtained images and recorded the distance between the index points. During the passive movement of the ankle joint and the toes by the flexion position and extension position, the point B determined by the resting position that moved with the passive movement was set as the “index point B’” and followed the cursor. At the specified, three still images were taken and saved, and the distance between the index points of “points A-B’“ was measured (Figure 5).

**Figure 6:**
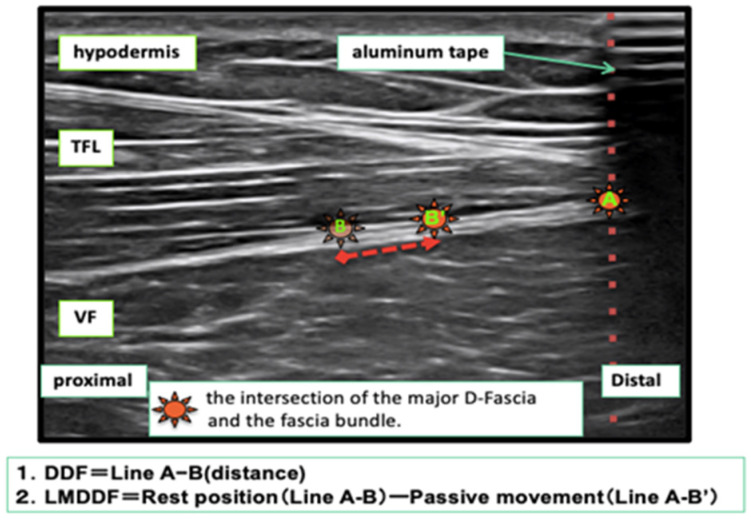
Measurement of the distance of deep fascia (DDF) movement of TFL.

To measure the length of the index point of the TFL, the aluminum tape was placed at the position where the shaded image of the aluminum tape was depicted in the distal part of the TFL depicted in the ultrasound image in resting position. The aluminum tape was used as the index point (point A). The probe was placed with respect to point A, and the landmark index point (point B) at the intersection of the D-fascia and the muscle fascicle within the muscle in the echo image depicting the TFL was taken, and still images were taken three times. We measured the length between the index points of “point A-B” obtained from these images. In the shooting during passive movement, the point D that moved with the passive movement was set as the index point B’ (point B’) and followed the cursor. Three still images were taken and the “point A-B’” distance was measured as the length between the index points (Figure 6).

The LMDDF was calculated from the difference between the distance between the index points of “point A-B” in resting position obtained from both G-med and TFL muscles and the distance between the index points of “point A-B’” obtained from flexion position and extension position photography. The average of the three measurements was adopted.

##### Measurement of Changes in Muscle Shape

**Figure 7:**
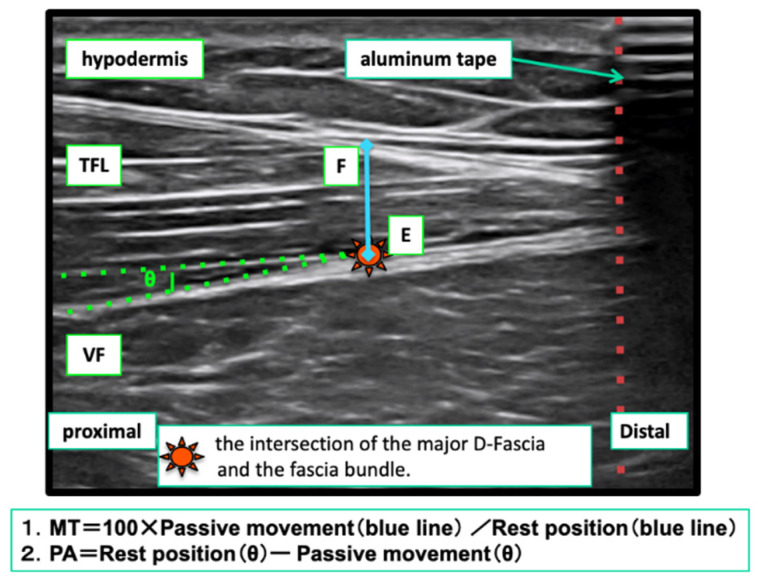
Shape change of muscle.

Using the method of Hodges P W [13] as a reference, the angle of the PA was deter- mined by the angle (∠θ) created by the intersection of the D-fascia and the muscle fascicle in the muscle, as depicted in the ultrasound image in resting position. Three still images were taken. After performing the passive task, the subjects followed the changing angle θ (angle θ’) and took and saved three still images when they reached the specified posture. In each passive task, the angle of the PA was measured three times and the average value was used. In the analysis, the angle θ was calculated from the difference between the angle θ at resting position and the angle θ’ obtained at flexion position and extension position (Figure 7).

For MT, the intersection of the pennation fascia in the muscle and the D-fascia in the deep layer was taken as the index point (point E), and a horizontal perpendicular line was drawn from point E. The intersection of the perpendicular line and the D-fascia on the most muscular tissue side of the D-fascia layer in the surface layer was taken as the index point (point F). After taking and saving three still images, we measured the length of the line between the index points (line E-F) (Figure 7). In addition, we followed the moving index points E and F (points E’ and F’) after the execution of the other action task, and took and saved still images three times when the posture was specified. MT was measured by drawing a line (line E’-F’) connecting the index points on the image. For each movement task, the index was measured three times and the average value was used. For analysis, the value of the line E-F with resting position as 100% is calculated. Subsequently, the rate of change of the line E’-F’ obtained from the flexion position and extension position shots was calculated and compared.

##### Surface Electromyogram Measurement

The sampling frequency of the surface EMG was set to 1 kHz/s, and the measured EMG waveform was filtered using a band-pass filter with a low pass filter of 20 Hz and a high pass filter of 500 Hz. Electromyogram (EMG) data were recorded during muscle rest and passive movement tasks of the toes and ankle joints. For data analysis, 3 s of stable EMG waveforms during the static and passive movement tasks were used. After full-wave rectification, the root mean square (RMS) of 3 s including the maximum EMG waveform was used. The RMS value of each muscle was the average value of the three measurements taken during each task. During the passive joint movement task, the EMG waveform was compared with the EMG waveform during resting position. The waveform was judged to be positive (expression of muscle contraction) when it exceeded twice the standard deviation of the static EMG waveform (2SD), referring to the method of Shimono [21] et al. If the EMG waveform was positive in a series of measurements, it was considered to be a case of induced muscle contraction of the measured muscle, and the ultrasound imaging data was excluded. Lab Chart (AD Instruments Inc., ver. 7) was used for data collection and analysis.

#### 2.2.6. Reliability Considerations

In order to examine the validity of the ultrasound measurement method, the reliability of the method was examined using the measurement results of 11 limbs of 11 subjects at the beginning of this study. To test the error range in the relative and absolute reliability of the measurement results, only the intra-examiner reliability was performed. The measurements were taken on the same day, and three measurements per task were taken twice with an interval of more than one hour. Eighteen items were measured for reliability, including the length between the index points of the D-fascia, the PA, and the MT, using the measurements of the three tasks of TFL and G-med.

### 2.3. Statistical Processing

Statistical measurements were performed using R-ver2.8.1. The rsting position and each passive task (flexion position and extension position) were subjected to a Shapiro–Wilk test to evaluate the normality of the data (distance between index points of the deep fascia, PA, and MT) obtained from each measurement item of G-med and TFL and the calculated values (LMDDF, change in PA, and rate of change in MT) of the data obtained from each measurement item.

Regarding the examination of the reliability of the measurement methods and values, the data obtained from each measurement item (length between index points of the D-fascia, PA, and MT) were subjected to ICC (1.1) to determine the relative reliability of the ultrasound imaging measurements performed in this study. In addition, because normality was observed in the length between index points of the D-fascia, PA, and MT of each muscle and each passive task, Bland–Altman analysis was performed to determine the type of measurement error and the range of error. The presence or absence of additive errors and proportional errors were confirmed. Then, when no systematic error was confirmed by Bland–Altman analysis, “MDC95” was calculated from the standard error of measurement [standard error of measurement: SEM] and “MDC” as an examination of the error range due to chance error (Equation (1)).

MDC95 = SEM × 1.96 × √2(1)

1.96: z-value of the 95% confidence interval

√2: Standard deviation of the sum of the variances of the two normalized groups of measurements

Next, in order to examine the amount of change and the rate of change of the measurement items, the calculation items “LMDDF, change of PA, and rate of change of MT” were tested for each of “resting position–resting position”, “resting position–flexion position”, and “resting position–extension position”. The Shapiro–Wilk test was used to check the normality of the calculated items. A nonparametric test was adopted because some of the corresponding calculated items showed normality while others did not. In order to examine the changes in the three parameters of TFL and G-med (LMDDF, change in PA, and rate of change in MT) during the resting position and passive joint movement (E and F), the Friedman test was used to analyze the difference between the three calculation items (difference in resting position–resting position, difference in resting position–flexion position, and difference in resting position–extension position), and the Bonferroni test was used for the post hoc test. For the rate of change in MT, the mean value of MT at the resting midpoint was set at 100%, and the rate of change in MT during each passive task was calculated and tested using that value. The significance level was set at *p* < 0.05.

## 3. Results

### 3.1. Surface Electromyogram Analysis

In one of the 21 subjects, an EMG waveform exceeding twice the SD of resting position was observed during the passive joint movement of the TFL.

Therefore, it was excluded from the ultrasound imaging measurement. Therefore, ultrasound measurements and analysis were performed on 20 limbs of 20 subjects for TFL and 21 limbs of 21 subjects for the middle fibers of the G-med.

### 3.2. Ultrasound Image Analysis

#### 3.2.1. Reliability Considerations

The measurement reliability, types of errors, and error ranges of the data (distance between index points of the deep fascia, PA, and MT) obtained for each measurement item of the TFL and G-med middle fibers of the G-med are shown in Table 2.

The intra-examiner reliability of the ICC using the TFL ultrasound system was 0.97 for the resting position, extension position, and flexion position for the distance between the index points of the D-fascia. The values for the PA were 0.74 for the resting position, 0.83 for the extension position, and 0.81 for the flexion position. For MT, the values were 0.96 for the resting position, 0.99 for the extension position, and 0.97 the for flexion position.

Bland–Altman analysis showed that there were no additive or proportional errors, only chance errors, in the TFL of “length between index points of D-fascia, PA and MT in ultrasound measurement” in this study. The error range of the length between the index points of the D-fascia from MDC95 to the resting position was 0.97 mm from the mean value, and the error range of the PA was 1.87° from the mean value. The error range of the MT was 0.32 mm from the mean value.

In the intra-examiner reliability ICC of the middle fibers of the G-med using ultrasound equipment, the distance between the index points of the D-fascia was 0.79 for the resting position, 0.88 for the extension position, and 0.79 for the flexion position. PA was 0.43 for the resting position, 0.69 for the extension position, and 0.68 for the flexion position. MT was 0.99 for the resting position, 0.95 for the extension position, and 0.99 for the flexion position. Bland–Altman analysis showed that there was no additive or proportional error in the distance between the index points of the D-fascia, but only chance error, and the error range of the length between the index points of the D-fascia in MDC95 was 2.12 mm from the mean value. An additive error was observed in the measurement of the extension position of the PA. A proportional error was also observed in the measurement of the resting position for MT.

#### 3.2.2. Results of Calculation Items

##### Results of TFL Calculation Items

**Figure 8:**
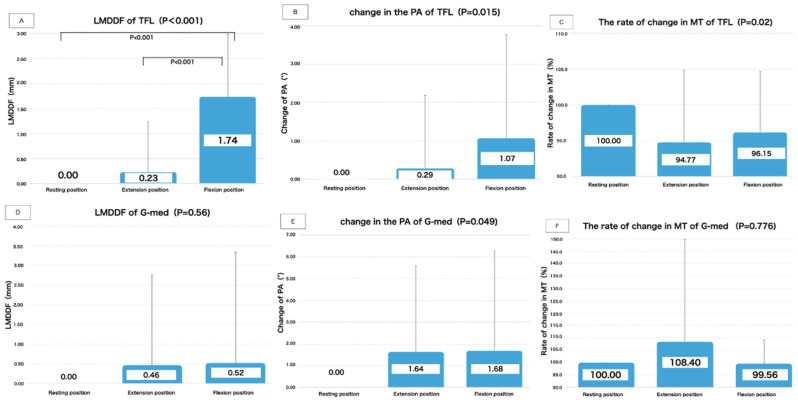
Results of each measurement item. (**A**,**D**) LMDDF and (**B**,**E**) change of PA calculated as the difference from resting position. Positive values of LMDDF indicate distance moved distal to the resting position measurement. (**C**,**F**) For the change in the angle of the PA, the positive value of the angle of the PA decreased compared to the value of the resting position measurement, indicating that the angle of the PA became more acute angle. The rate of change in MT is shown when the MT length at resting position is set to 100%.

The results of LMDDF, change of PA, and percentage change in MT of the TFL are shown in Table 3 and Figure 8.

In TFL, there was a significant change in the LMDDF between the resting position, extension position and flexion position (*p* < 0.001). A significant main effect was observed in the deep fascia movement on the TFL associated with passive movements of the toes and ankle joints. The results of the multiple comparison (Bonferroni) test showed a significant difference (*p* < 0.001) in the distance of movement from resting position to flexion position. Furthermore, significant mobility of the D-fascia was confirmed between the distance of movement of the extension position and flexion position (*p* < 0.001). During the passive movement of the ankle joint and toes from the resting position to flexion position, a mean D-fascia mobility of 1.74 mm (SD 1.25) was observed in the distal direction of the lower extremity compared to the resting position measurement.

The difference between the two levels was not significant for the change in the PA and the percentage change in MT in TFL.

##### Results of G-Med Calculation Items

There were no statistically significant changes between the three levels in resting position, Extension position, and flexion position for “LMDDF, PA, and percentage change in MT” in G-med. (Table 3 and Figure 8.)

## 4. Discussion

### 4.1. Summary of Research and What Is New

In order to investigate the functional role of the continuity of the fascial tissues of the lower extremity, as evidenced by the anatomical studies by Stecco A [5] and Wilk J [6], and the response to mechanical tension, the presence or absence of mobility and changes in muscle shape of the D-fascia of the G-med and TFL during passive movements of the toes and ankles were investigated in this study. We actually observed the inactive state of the muscle using electromyography, and confirmed the mobility of the deep fascia of the TFL by passive movement of the ankle joint, even though no muscle contraction of the TFL occurred.

In this study, we first examined the reliability of the ultrasonic measurement method, and determined the error range in resting position by calculating the MDC95 during resting position for each measurement item of “distance between index points of deep fascia, PA, and MT.” In the calculation items between the resting position and the passive movement, such as “LMDDF, change in PA, and rate of change in MT,” true change was judged to have occurred only when the calculation results during passive movement of the ankle joint and toes exceeded the MDC95 of the resting position.

As a result, in TFL, the LMDDF in the distal direction of the lower limb in flexion of the toes, plantar flexion of the ankle joint, and inversion position (flexion position) (mean 1.74 mm, SD 1.25) was higher than the value of MDC95 (0.97 mm) during the resting position of the toes and ankle joint, confirming that there was a change beyond error. There was also statistically significant mobility of the D-fascia of the TFL in the distal direction during the flexion position compared to the resting position of the toe and ankle joint. Significant changes were also observed in the LMDDF in TFL during toe extension, ankle extension and valgus position (Extension position) and toe flexion and the ankle flexion position. This result partially supported the hypothesis of this study, “Mobility of the D-fascia and changes in muscle shape are observed in both TFL and G-med during passive movement of the toes and ankle joints”.

This study is the first to elucidate in vivo the transmission of mechanical tension caused by the continuity of lower limb fascial tissue from the distal to the proximal portions, with respect to the results of previous cadaveric autopsy studies that demonstrated the anatomical connectivity of the fascia.

### 4.2. For Each Parameter of the TFL

As for the LMDDF, the flexion position of the toe and ankle joint showed significant mobility in the distal direction of the lower limb compared to other passive tasks. This is thought to be due to the transmission of tension from the collagen tissue, which is the main component of the D-fascia, and tension from fascial expansion to the proximal part. The term “fascial expansion” refers to the respective connections derived from skeletal muscles or their tendons, tendons that insert into the aponeurotic fascia [22]. These developments were usually considered as anatomical breakthroughs, but all were found to exhibit precisely constant organization. Huijing P A et al. [23] also argued that 30–40% of the tension that occurs in the muscle is propagated by the connective tissue surrounding the muscle, rather than along the tendon.

In terms of anatomical findings, a study by Stecco A et al. [5], which showed the anatomical and functional relationship between G-max and TFL, confirmed that G-max is connected to the iliotibial ligament, femoral fascia, and TFL, and proved that the femoral fascia provides a small origin from multiple muscles of the thigh, explaining the transmission of force from G-max to the knee periphery.

Wilke J [6] also reported the connection between the iliotibial ligament and the peroneus longus fascia, and estimated the mechanical connection between the two.

In addition, Stecco L [24] described the arrangement of the D-fascia in the lateral portion of the lower extremity, from the G-max and TFL to the extensor digitorum longus and peroneus longus via the vastus lateralis, lateral retinaculum of patella, and iliotibial ligament.

The results of this study confirmed the mobility of the D-fascia in the distal direction of the lower limb in the passive compound flexion position of the toe and ankle joints, and estimated the tension transmission of the muscle tissues of the distal lower limb such as the peroneus longus and extensor digitorum and the D-fascia covering them due to the anatomical connection between the fascia and the muscle tissues.

The fascia transmits the tensile tension generated by the muscle through its unfolding, and this tension is propagated across the joint by the fascia.

The physiological function is that the contraction and extension of the proximal muscle is changed through the firing of the distal muscle spindle in the fascia [25].

Langevin H M et al. [26,27] and Benjamin M et al. [28] reported that endogenous fibroblasts in fascia are essential for mechanical signaling and that they communicate with each other via gap junctions and respond to tissue elongation caused by cytoskeleton-mediated shape changes. They reported that the cytoskeleton responds to tissue elongation caused by cytoskeleton-mediated shape changes.

These results suggest that the D-fascia of the TFL moved in the distal direction of the lower extremity by transmitting tension through the D-fascia to the lower leg fascia, iliotibial ligament, and femoral fascia during the flexion position of the toe and ankle joint.No significance was found in the comparison of the PA of each task. In this method of measuring the PA, the reference axis was the deep fascia of the TFL adjacent to the muscle spanning the lower part (vastus lateralis), and the axis of movement was the PA within the muscle of the TFL. Therefore, during passive movement of the toe and ankle joint, muscle shape changes were observed not only for the TFL but also for the vastus lateralis muscle spanning the lower part. Anatomically, the vastus lateralis muscle fibers have extensive attachments to the lateral intermuscular septum and the lateral retinaculum of patella, and the iliotibial ligament also has extensive attachments to the lateral retinaculum of patella [24,25,29,30].

Therefore, it is thought that the tension response of the D-fascia occurs from the lower leg fascia to the femoral fascia during the other movements of the toes and ankle joints, and that the iliotibial ligament transmits tension and changes muscle shape to the muscle fibers of the vastus lateralis muscle or the D-fascia through the lateral interfascicular septum and lateral retinaculum of patella. In addition, it is possible that the passive movement of the toe and ankle joint caused changes in muscle shape not only in the muscle fascicle within the muscle of the TFL but also in the vastus lateralis muscle, thereby affecting the D-fascia tissue of the TFL, which is the basic axis for measuring the angle of the vastus lateralis muscle.

Hodges P W [13] previously reported a study of muscle shape change using MT and PA change as parameters, and the basic axis of measurement in that study was bone as an index where no morphological change occurred. In this study, the soft tissue (D-fascia) was treated as the basic axis, so the D-fascia response of fascia in the passive movement may have spilled over to the vastus lateralis muscle lying below the TFL, and no statistical change was observed. It is necessary to continue to study the measurement method.

### 4.3. For Each Parameter of the G-Med

There were no significant changes in the G-med “LMDDF, change in the angle of PA, and rate of change in MT” in comparison with each exercise item. As for the criteria for ICC, Landis [31] defined 0.81–1.00 as almost perfect, 0.61–0.80 as substantial, 0.41–0.60 as moderate, 0.21–0.40 as fair, and 0.0–0.20 as slight. The results for MT were almost perfect, but the results for the length between the index points of the deep fascia were substantial, and the results for the PA were moderate.

First of all, the G-med is located below the adjacent gluteus maximus muscle compared to the TFL, so it is prone to errors due to the way the probe is applied in the diagnostic ultrasound evaluation of in vivo measurements. In addition, in the measurement of G-med, not only a chance error, but also an additive, systematic and proportional error existed. Therefore, there is still room for further study on the measurement of G-med, including the measurement method.

Second, the subject was placed in a side-lying position, and the measurement was performed in the intermediate position of hip flexion and extension, and in the intermediate position of internal and external rotation. Compared to the TFL, which has a hip flexion action, the middle fibers of the G-med used in this G-med measurement are hip extensors, and the middle fibers of the G-med take a relatively shortened position in the mid-hip position. In the measurement position, G-med may have taken a limb position in which the tension transmission of the deep fascia was less effective than that of TFL in the stretched position.

### 4.4. Clinical Application

In this study, we were able to partially demonstrate in vivo the continuity of the fascia connecting skeletal muscle to skeletal muscle in vitro and the tension response of the D-fascia, which has been reported in previous papers. There has been no previous study demonstrating the transmission of tension by fascia across multiple joints in vivo. This paper will contribute to the accumulation of knowledge in the field of biological structure, kinematics, and anatomy as basic research on fascia in vivo, and will enable the development of research in the fields of manual treatment of fascia in vivo, pathological analysis, and functional evaluation. The results of the present study demonstrated the transmission of force across two or more joints by fascia. This suggests, for example, that contractures in the soft tissues of the foot may also affect the tension in the soft tissues of the hip joint.

### 4.5. Research Limitations

The present study has research limitations. The first limitation is that we did not measure the parameters for women because the subjects were healthy adult males, and the measurement results may change due to differences in fat mass, muscle mass, and skeletal structure. In this regard, it is necessary to take gender differences into account in the measurements.

Second, it does not take into account individual differences in muscle tightness and muscle length. The D-fascia, including the epimysium fascia and the aponeurotic fascia, has a close connection with the muscle tissue and propagates the muscle tension to the D-fascia. Therefore, it may affect the measured values for subjects with muscle tightness and cross-link formation of muscle collagen fibers.

Third, these results may not be similar to those obtained for other muscles and joints. It is necessary to continue measurements to clarify the function and structure of fascia not only in the lower limbs but also in the trunk and upper limbs.

## 5. Conclusions

With the inactivity of the muscles in the middle fibers of the G-med and TFL monitored by surface electromyography, the LMDDF and changes in muscle shape of both muscles during passive movements of the toes and ankle joints were investigated. Compared with the resting position, the D-fascia of the TFL moved significantly in the distal direction of the lower extremity during the passive movement of the flexion position in flexion of the toe and ankle joint and in inversion position. It was suggested that the passive stretch applied to the peripheral muscles of the toes and ankle joints and the D-fascia may be transmitted to the fascia of the proximal muscles, which are farther away due to the anatomical continuity of the fascia.

## Figures and Tables

**Table 1:** Basic physical data of the test subjects.

Item	Mean (SD)
gender	21 males
Age	24 years old (±4.1)
Height	171.8 cm (±6.43)
Weight	66.9 kg (±7.15)
BMI	22.7 (2.71)
ROM of Ankle joint	
Dorsiflexion	17.9° (±5)
Plantar flexion	45.8° (±3.35)
Horizontal angle of test limb	0.28°(±0.21)

**Table 2:** Results of ICC and Bland–Altman analysis for each measurement item.

**Measured (1st Mean vs. 2nd Mean) *n* = 11**									
**Reliability of TFL**									
**Item**	**Limb Position**	**ICC(1, 1)**	**Additive Error**		**Proportional Error**		**MDC** **(Minimal Detectable Change) {mm}**	**Standard Error (SE)**	**MDC95**
			**95% CI of the Mean of the Differences**	**Yes or No**	**Testing for Correlation Dominance**	**Yes or No**			
LMDDF	Resting	0.97	−0.79~0.77	no	*p* = 0.15	no	2.28	0.35	0.97
	extension	0.97	−0.81~0.55	no	*p* = 0.2	no	1.97	0.30	0.84
	flexion	0.97	−1.00~0.61	no	*p* = 0.76	no	2.36	0.36	1.01
PA	Resting	0.74	−1.79~1.21	no	*p* = 0.87	no	4.38	0.67	1.87
	extension	0.83	−0.48~1.47	no	*p* = 0.54	no	2.84	0.44	1.21
	flexion	0.81	−1.20~1.72	no	*p* = 0.06	no	4.26	0.66	1.82
MT	Resting	0.96	−0.08~0.44	no	*p* = 0.1	no	0.76	0.12	0.32
	extension	0.99	−0.12~0.13	no	*p* = 0.71	no	0.37	0.06	0.16
	flexion	0.97	−0.19~0.32	no	*p* = 0.64	no	0.74	0.11	0.31
**Measured (1st Mean vs. 2nd Mean) *n* = 11**									
**Reliability of G-Med**									
**Item**	**Limb Position**	**ICC(1, 1)**	**Additive Error**		**Proportional Error**		**MDC** **(Minimal Detectable Change) {mm}**	**Standard Error (SE)**	**MDC95**
			**95% CI of the Mean of the Differences**	**Yes or No**	**Testing for Correlation Dominance**	**Yes or No**			
LMDDF	Resting	0.79	−1.03~2.39	no	*p* = 0.87	no	4.98	0.77	2.12
	extension	0.88	−1.65~1.31	no	*p* = 0.97	no	4.32	0.67	1.84
	flexion	0.79	−0.33~2.84	no	*p* = 0.07	no	4.63	0.71	1.98
PA	Resting	0.43	−2.37~5.16	no	*p* = 0.33	no	10.99	1.69	4.68
	extension	0.69	0.36~4.42	yes	*p* = 0.46	no	5.93	0.91	2.53
	flexion	0.68	−1.15~4.05	no	*p* = 0.41	no	7.59	1.17	3.23
MT	Resting	0.99	−0.19~0.17	no	*p* = 0.05	yes	0.52	0.08	0.22
	extension	0.95	−1.20~0.12	no	*p* = 0.63	no	1.92	0.30	0.82
	flexion	0.99	−0.55~0.21	no	*p* = 0.45	no	1.12	0.17	0.47

**Table 3:** Results of the LMDDF, change of PA, and rate of change in MT for each muscle.

Item	TFL (*n* = 20)		G-Med (*n* = 21)	
	LMDDF (Difference: mm)	*p* Value	LMDDF (Difference: mm)	*p* Value
	Mean (±SD)	*p* < 0.001	Mean (±SD)	*p* = 0.56
Resting-Extension	0.23 (1.01)	0.4	0.46 (2.29)	
Flexion-Extension	1.51 (0.12)	0.001	0.059 (0.26)	
Resting-flexion	1.74 (1.25)	0.001	0.52 (2.81)	
	Change of PA	*p* value	Change of PA	*p* value
	Mean (±SD)	*p* = 0.015	Mean (±SD)	*p* = 0.049
Resting-Extension	0.29 (1.91)	0.5	1.64 (3.95)	0.378
Flexion-Extension	0.78 (0.40)	0.4	0.04 (0.33)	1
Resting-flexion	1.07 (2.70)	0.089	1.68 (4.6)	0.086
	rate of change in MT (%)	*p* value	rate of change in MT (%)	*p* value
	Mean (±SD)	*p* = 0.02	Mean (±SD)	*p* = 0.776
Resting-Extension	94.77 (10.08)	0.433	108.4 (41.59)	
Flexion-Extension	※98.56 (0.77)	0.064	※108.88 (15.94)	
Resting-flexion	96.15 (8.54)	0.088	99.56 (9.72)	

Changes in LMDDF and PA are calculated as the difference from resting and the difference from extension to flexion. A positive value for LMDDF indicates a distance moved more distally than the measured value for resting. In the case of the difference from extension to flexion, a positive value indicates that flexion has moved more distally than extension. A positive value for PA indicates that PA has become more acute angle. A positive value for the difference of flexion from extension indicates that flexion became more acute angle than extension. The rate of change of MT is shown when the length of muscle thickness of resting is 100%. The rate of change from flexion is shown.

## Data Availability

Not applicable.
